# Introducing the Dilation and Evacuation Technique in Brazil: Lessons Learned From an International Partnership to Expand Options for Brazilian Women and Girls

**DOI:** 10.3389/fgwh.2022.811412

**Published:** 2022-02-22

**Authors:** Bianca M. Stifani, Susane Mei Hwang, Renata Rodrigues Catani, Helena Borges Martins da Silva Paro, Nerys Benfield

**Affiliations:** ^1^Department of Obstetrics & Gynecology, New York Medical College, Valhalla, NY, United States; ^2^Department of Obstetrics & Gynecology, Albert Einstein College of Medicine, Bronx, NY, United States; ^3^Hospital Maternidade Vila Nova Cachoeirinha, São Paulo, Brazil; ^4^Department of Obstetrics & Gynecology, Universidade Federal de Uberlândia, Uberlândia, Brazil

**Keywords:** abortion (induced), dilation and evacuation, capacity building, clinical simulation and skills, surgical training, Brazil

## Abstract

Dilation and evacuation (D&E) is the recommended surgical procedure for uterine evacuation in the second trimester. Despite its established safety record, it is not routinely available in most countries around the world. In this paper, we describe the multi-phase capacity-building project we undertook to introduce D&E in Brazil. First, we invited a highly motivated obstetrician-gynecologist and abortion provider to complete an observership at an established D&E site in the United States. We then organized a month-long clinical training for two experienced gynecologists in Brazil, followed by ongoing remote mentorship. Almost all patients we approached during the training opted for D&E, and all expressed satisfaction with their experience. Despite the restrictive legal setting and prevailing abortion stigma in Brazil, our training was well-received, and we did not experience any overt resistance from hospital staff. We learned that obtaining institutional support is essential; and that presenting scientific evidence during dedicated didactic times was an important strategy to obtain buy-in from other local healthcare providers. An important challenge we encountered was low case volume given the restrictive legal setting. We addressed this by partnering with nearby hospitals and non-profit organizations for patient referrals. We also rescheduled, adapted and optimized this project for implementation in the midst of the COVID-19 pandemic. Despite the challenges we faced, this project led to the successful introduction of D&E up to 16–18 weeks at two sites in Brazil. In the future, we plan additional training to increase capacity for D&E at more advanced gestational ages.

## Introduction

In the 1970s, physicians in England and the United States introduced a surgical technique for uterine evacuation in the second trimester of pregnancy (1, 2). This technique, known as dilation and evacuation (D&E), has replaced hysterotomy and hysterectomy and is now the only method recommended by the World Health Organization (WHO) for surgical abortion beyond 12–14 weeks (3). D&E serves as an important alternative for patients who have contraindications to or have failed labor induction, or for those who prefer a surgical procedure instead. Unlike for medical abortion, there are no absolute contraindications to D&E (3). The D&E technique is most used in the context of induced abortion, but it can also benefit patients with intrauterine fetal demises (IUFD) or pre-viable preterm premature rupture of membranes (PPROM) (4, 5).

Compared to labor induction, D&E is faster, has a lower incidence of minor and major complications, and fewer cases of retained products (6–9). In cases of fetal anomalies or fetal death, one study described an adjusted risk ratio for any complication of 8.5 (9.5%CI 3.7–19.8) for labor induction over D&E (4).

Despite the established safety record for D&E, this procedure has only been described in a handful of countries (United States of America, United Kingdom, Australia, France, Netherlands), and is not routinely available in most countries around the world (2). One study described the first 400 D&E cases performed after the procedure was introduced in Vietnam, but its authors did not explain in detail how they trained providers and introduced the service (10).

In Brazil, a country of more than 210 million people, there were no trained D&E providers when we undertook this project. Women and girls, many of whom are rape victims, often undergo lengthy and complicated inductions, which at times even result in hysterotomy if the induction fails. In response to the pressing need to expand options for second trimester uterine evacuation for Brazilian women and girls, we established an international partnership to introduce D&E in Brazil. Here, we describe this multi-phase capacity-building project and analyze the challenges and successes we encountered as we implemented it in the midst of the COVID-19 pandemic. We highlight lessons learned and provide recommendations for others wishing to introduce D&E services in new settings.

## Context and Description of Three-Phase Project

### Phase 1: Clinical Observership at Established D&E Site

In August of 2019, one of the authors (HP), a Brazilian OB-GYN, contacted the Family Planning Division of the Albert Einstein College of Medicine/Montefiore Medical Center in New York, USA. She leads a clinical service for women and girls who are victims of sexual violence in the state of Minas Gerais. Her clinic is the only site that provides legal abortions in a region of more than 2 million people. Many of her patients are adolescents, who often present in the second trimester due to delays in recognizing pregnancy, difficulties in accessing care, and lack of knowledge about their right to abortion. Because D&E was not available anywhere in Brazil, the only option for abortion beyond 12 weeks was a labor induction in the hospital's obstetric ward. Having observed a number of lengthy, traumatic, and even failed inductions, HP felt compelled to seek D&E training in order to offer this alternative to her patients. She completed a funded, month-long observership at Albert Einstein/Montefiore, during which she observed ~20 D&Es. She also observed the corresponding pre-operative visits and learned about the necessary testing, consent procedures, surgical instruments, and follow-up. However, she was not able to obtain hands-on training due to hospital regulations.

### Phase 2. Focused Clinical Training Course at Local Site

Following HP's observership, we sought funding for and organized a four-week clinical training in Brazil. We partnered with Hospital Maternidade Vila Nova Cachoeirinha in São Paulo. The objective was to train HP and another OB-GYN from Cachoeirinha Hospital (SH), to become competent in providing D&E in the early second trimester. We also sought to teach OB-GYN residents and other attending physicians about the fundamentals of D&E. We therefore organized a six-lecture series covering clinical topics related to D&E as well as socio-legal topics such as abortion in Brazil and sexual violence. We held the lectures in Portuguese and in hybrid format (in person but broadcast via Zoom for those who could not attend), and also invited interested physicians from other hospitals around Brazil. On average, 40 people attended each lecture.

**Figure 1:**
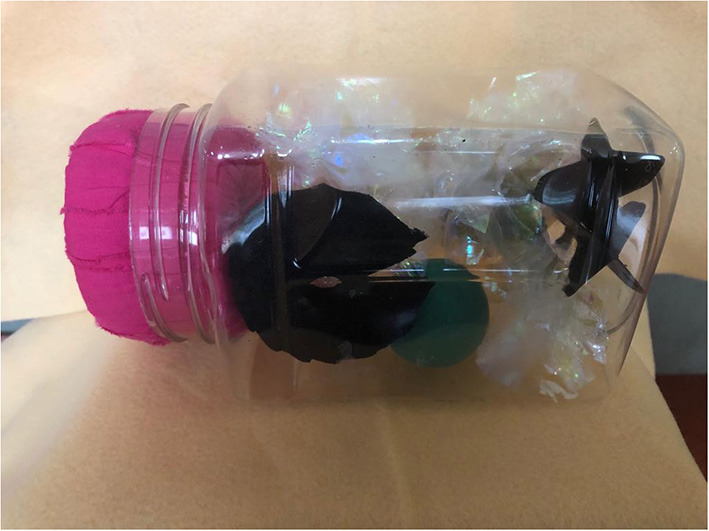
D&E simulation model made with 32 oz plastic container as uterus; mylar balls and flexible darts as “fetal parts”.

**Figure 2:**
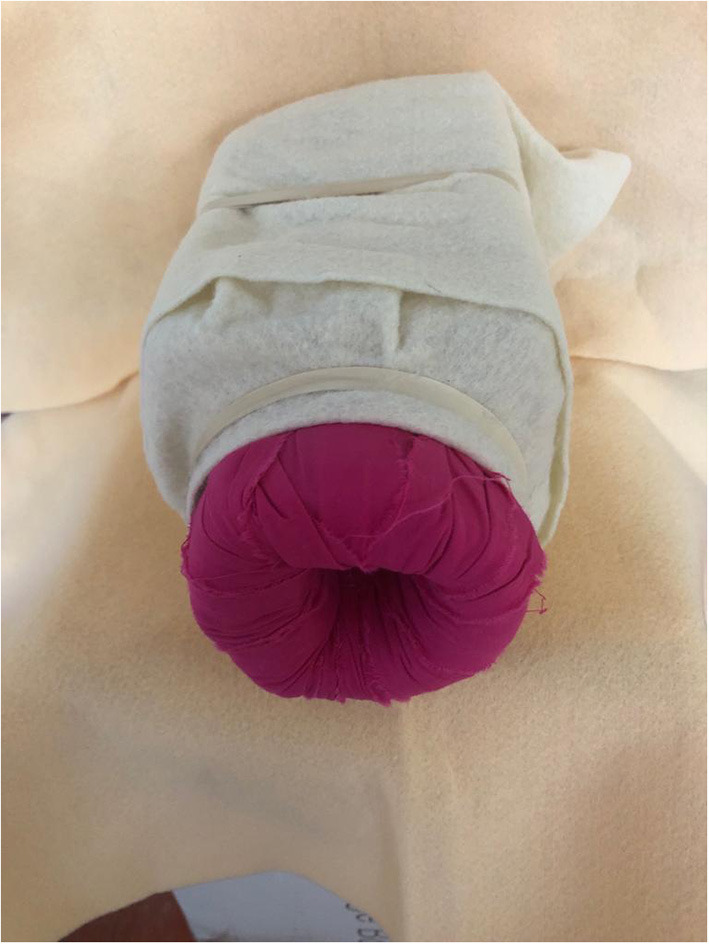
D&E model covered with felt to practice extraction of “fetal parts” by feel.

**Figure 3:**
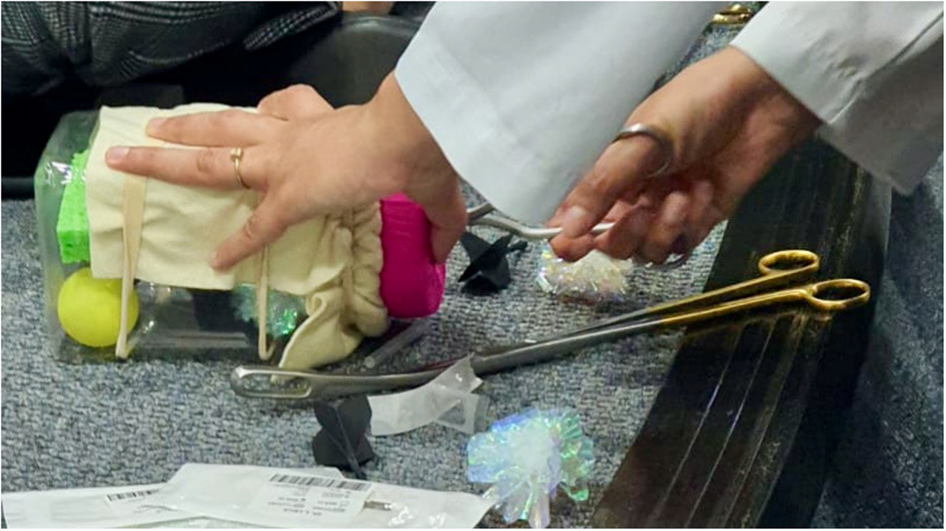
D&E model in action with Bierer forceps used to extract “fetal parts”.

We also organized a D&E simulation for local physicians utilizing a low-cost, low-fidelity simulation model similar to those described by Baldwin and Chor (11, 12). The model consists of a “uterus” made of a 36 oz plastic rice jar and a “cervix” made using foam can holders cut and glued to different cervical “dilations,” then covered with fabric. We used a felt fabric square to hide the contents of the “uterus:” “fetal parts” which were cat toys (mylar balls) and flexible darts (see Figures 1, 2). Simulation participants used the foam cervices to practice performing paracervical blocks and used Bierer and Sopher forceps to remove “fetal parts” from the uterus (see Figure 3).

For the clinical component of the training, we approached patients who were seeking legal abortions from the Hospital's service. These were either cases of rape or anencephaly. We approached patients with gestational ages of 13 weeks and above as these cases would previously have been managed medically (the largest available suction cannula is 12 mm). We also approached patients with obstetrical diagnoses such as intrauterine fetal demise or PPROM and offered the procedure. We explained to patients that we were introducing D&E in Brazil and that they could choose this procedure or proceed with labor induction, which was the standard of care at Cachoeirinha Hospital. We explained the risks and benefits of each option, and also explained that a learner (HP or SH) would be performing the procedure alongside an expert physician (BS). Patients who chose D&E signed an informed consent form and underwent cervical preparation as clinically indicated. For patients under 14 weeks we used misoprostol alone for cervical preparation, while patients 14 weeks or above received cervical dilators.

We obtained approval from the Hospital's Ethical Committee for all components of the training, and from the Regional Medical Council for BS to practice medicine in a limited capacity in São Paulo.

During this phase of the project, we approached 13 patients, and 12 of them chose D&E over labor induction. One delivered before we could perform the D&E. We performed 11 D&Es for patients with gestational ages ranging from 13 to 21 weeks (see Table 1 for a breakdown of clinical characteristics for each case). We had two complications (cervical laceration requiring repair, and excessive bleeding requiring uterotonics but not transfusion), and no serious complications. We performed all D&Es under ultrasound guidance with RRC as ultrasonographer.

**Table 1:** Clinical characteristics of D&E cases performed during focused clinical training period at Hospital Maternidade Vila Nova Cachoeirinha, São Paulo.

**Case**	**Age**	**GA**	**Indication**	**Obstetric history**	**Comorbidities**	**Complications**
1	27	18 weeks 5 days	Fetal demise^*^	VD x1	None	None
2	14	21 weeks 0 days	Rape	None	Teen pregnancy	Cervical laceration requiring repair
3	27	15 weeks 0 days	Rape	CD x 2	Obesity	None
4	21	16 weeks 1 days	Rape	CD x 2	Sickle cell trait	None
5	31	17 week 2 days	Rape	VD x 1	Placenta previa	None
6	27	15 weeks 3 days	Fetal demise^*^	CD x 2	None	None
7	23	20 weeks 1 day	Rape	None	None	None
8	37	16 weeks 6 days	Rape	VD x 6	None	None
9	31	13 weeks 1 days	Anencephaly	VD x 2	None	None
10	23	13 weeks 2 days	Rape	None	Placenta previa	Bleeding requiring uterotonics
11	21	15 weeks 0 days	Anencephaly	None	None	None

* *For fetal demises we present the size of the fetus rather than the GA by dates*.

*GA, gestation age; VD, vaginal delivery; CD, cesarean delivery*.

### Phase 3. Ongoing Clinical Support & Follow-Up Training

After the four-week focused training period, BS remained available for remote consultation and support. SH has since performed nine additional D&Es at Cachoeirinha Hospital, with gestational ages ranging from 14 weeks 2 days to 18 weeks. HP has performed two D&Es at 15 weeks in Uberlândia. Table 2 shows the clinical characteristics for each of these cases, which were uncomplicated. In the future, we plan to provide additional clinical training at more advanced gestational ages. This will require that an experienced D&E provider be available in São Paulo for a longer period of time. Another future goal is for HP and SH to train additional interested providers once their comfort level with performing the procedure further increases. This will eventually lead to the procedure becoming available at other sites throughout Brazil.

**Table 2:** Clinical characteristics of D&E cases performed independently by newly trained providers after focused clinical training.

**Case**	**Age**	**GA**	**Indication**	**Obstetric history**	**Comorbidities**	**Complications**
1	21	15 weeks 0 days	Anencephaly	None	None	None
2	39	16 weeks 3 days	Rape	None	Obesity	None
3	25	14 weeks 5 days	Rape	None	None	None
4	27	16 weeks 4 days	Rape	VD x 2	None	None
5	18	15 weeks 4 days	Rape	None	None	None
6	23	14 weeks 2 days	Rape	None	None	None
7	21	15 weeks 0 days	Anencephaly	CD x 1	None	None
8	25	15 weeks 1 days	Anencephaly	None	None	None
9	34	16 weeks 2 days	Rape	None	None	None
10	36	18 weeks 0 days	Risk to life	CD x 1	Placenta previa; placental abruption	None
11	33	15 weeks 6 days	Anencephaly	VD x 1	None	None

*GA, gestation age; VD, vaginal delivery; CD, cesarean delivery*.

## Challenges Encountered

### Case Volume in Restrictive Legal Setting

The grounds for legal abortion in Brazil are limited to cases of rape, anencephaly, or risk to a woman's life (13). Further, many of those who would be eligible for legal abortion are not aware of their rights and do not seek care. Therefore, relatively few legal abortions take place in Brazil (14), and we knew that a successful focused clinical training would have to take place in one of the country's busiest abortion services. We also knew that we could only train one or two providers, although more had expressed interest in learning this skill. We therefore partnered with Cachoeirinha Hospital, where, on average, 3–4 patients seek abortion in the second trimester each week. We also informed service providers from nearby hospitals and advocates from a national NGO that the training would be happening during specific dates and that they could refer patients from the region to Cachoeirinha for D&E. Several patients did come as referrals from nearby hospitals and the NGO, but despite our efforts we did not reach our initial goal of 20 D&Es for the four-week focused training period. However, given that HP and SH are experienced gynecologic surgeons, the number of cases was sufficient for them to feel comfortable performing D&E independently to 16–18 weeks. Although we did not use an objective strategy to assess competency, BS is an expert D&E provider and trainer and was able to assess HP's and SH's competency. To provide additional training for more advanced gestational ages, we are planning for an experienced D&E trainer to spend a longer period of time in São Paulo in the near future.

### Obtaining Surgical Instruments and Other Supplies

Surgical instruments for D&E and dilators for cervical preparation were not available at Cachoeirinha Hospital. We initially attempted to purchase the surgical instruments in Brazil but no company was authorized to import them and it was not possible to purchase them from Brazil. We therefore purchased the instruments in the United States and shipped them to Brazil. This was a quick but expensive endeavor given high importation and customs fees. We also shipped laminaria, which are not commercially available in Brazil. We were able to purchase Dilapan-S in Brazil. Other necessary equipment and supplies were already available at the hospital, such as an ultrasound machine for intraoperative guidance; manual vacuum aspirators (MVAs); and suction cannulas. We purchased spinal needles for paracervical blocks in Brazil and obtained syringes and injectable anesthetic agents through the hospital. After the focused training ended, Cachoeirinha Hospital experienced a shortage of cervical dilators until SH received a donation of additional Dilapan-S. In the future, should there be difficulties in purchasing additional dilators, cervical foley with serial misoprostol will be a possible alternative for cervical preparation (15).

### Delays and Adaptations in the Context of the COVID-19 Pandemic

Phase 2 of the project was originally scheduled for August of 2020 but had to be rescheduled due to COVID-19 infection risk and travel restrictions. We rescheduled it for the Spring of 2021, once COVID-19 vaccines became available and institutional and governmental travel restrictions were lifted. We adapted the training in two ways in response to the pandemic: first, we canceled a planned component that included outside trainers traveling to Sao-Paulo to conduct in-person workshops to explore attitudes about abortion; second, we held lectures in hybrid format and with fewer people present in person. Because none of the procedures we performed were considered elective, we did not have issues scheduling them or accessing the operating rooms. Further, the hospital did not require preadmission COVID-19 testing in asymptomatic patients and we therefore did not experience delays from that. At the time of the training (May–June 2021), the COVID-19 incidence rate in Brazil was extremely high. However, Cachoeirinha Hospital is exclusively a maternity hospital, and although several pregnant patients were admitted in critical condition due to COVID-19 pneumonia during our training, the hospital was not at capacity. Our anesthesia team did request that we perform procedures under regional anesthesia with minimal sedation rather than moderate or deep sedation to avoid the need to ventilate patients due to a concern for possible increased COVID-19 transmission. Finally, BS did bring personal protective equipment from the United States to avoid straining the hospital's supply.

## Successes

### High Patient Acceptance and Satisfaction With the Procedure

Acceptance of the D&E procedure among patients was extremely high. Of the 13 patients we approached during our focused clinical training period in São Paulo, 12 opted for D&E over labor induction. Although we did not conduct formal patient satisfaction surveys, we received positive feedback from all patients who had D&Es, who were highly satisfied with the procedure and grateful for its availability.

### Overwhelming Support From Hospital Leadership and Other Providers

In Brazil, abortion stigma is rampant, and the anti-abortion movement has gained momentum since the election of President Bolsonaro. Abortion advocates and providers are routinely harassed and at least one lives in exile because she received multiple death threats (16, 17). Despite this challenging sociopolitical environment, our training proposal was met with overwhelming support from the hospital's leadership, and we did not sense any overt resistance or hostility from other physicians or staff at the hospital. We approached the hospital's director with a thorough proposal in which we outlined the possible indications of D&E as well as its risks and benefits in comparison with labor induction. We also submitted the proposal to the hospital's ethics committee and were available to answer any of the board members' questions. Although some initially asked for clarification on specific issues, the proposal was unanimously approved by the committee as its members recognized the potential benefits to the hospitals' patients.

### Clinical Adaptations

We did adapt our clinical practice to some extent given the relatively resource-limited setting. First, because mifepristone is not available in Brazil, we did not have this as an option for cervical ripening. Second, the hospital did not have an electric vacuum aspiration machine or tubing. We therefore used manual uterine aspirators during D&E (using a 12 mm suction cannula, which was the largest available). Third, we performed the procedures under spinal anesthesia with minimal sedation per the preference of our anesthesia team. Finally, given that many of the patients lived far from the hospital and did not have access to transportation in case of overnight emergencies, we admitted all patients who required cervical preparation to the labor and delivery unit overnight prior to D&E. This did not add additional burden to the hospital staff as these patients would otherwise have been admitted to the hospitals for medication abortion.

## Discussion: Lessons Learned, Recommendations for Other Global Health Practitioners, and Limitations

Lessons Learned:

(a) Plan focused training at highest possible volume site and seek referrals from surrounding areas. Case volumes can be unpredictable, particularly if the legal grounds for abortion are limited; it is therefore important to plan short trainings at the highest volume sites and to seek referrals from surrounding areas. Ultimately, case numbers may be insufficient for providers to reach competency during shorter training periods and repeated trainings may be required.
(b) Use limited training time to train one or two providers only. Given that case volume is a rate-limiting step for focused D&E training, it is imperative to select only one or two highly motivated providers for the initial training, setting the expectation that once competent, they will train others who are interested in acquiring the skillset.
(c) Train providers who are experienced gynecologists and who are highly motivated to acquire and immediately apply the D&E skillset. Although HP and SH completed relatively few proctored cases, they were able to demonstrate competency in D&E in the early second trimester as they already possessed an advanced skillset in procedural gynecology. They also were motivated to continue improving their skills by offering this service to their patients immediately after the training.
(d) Obtain buy-in from hospital leadership and necessary authorities. Although the process for seeking approvals and authorizations may vary in different countries and setting, obtaining buy-in from people in leadership roles is key. We found that presenting the scientific evidence to a group of physicians (of various specialties) was sufficient to convince them of the value of our proposed training.
(e) Provide high quality clinical lectures and simulation for other interested providers. Although we could only provide clinical training to two physicians, we prepared a lecture series for other interested physicians. The focus of the lectures was on presenting the scientific evidence and explaining the technique. We found that the lectures were well-attended, and the materials were well-received. Attendees had the opportunity to ask questions, and even those who were skeptical about D&E were able to engage in productive conversation. The simulation was an opportunity for residents to see the surgical instruments and familiarize themselves with certain aspects of the procedures. This was also well-received and well-attended.
(f) Consider providing exposure to an established D&E site. HP's observership at an established D&E site allowed her to have additional exposure to the procedure and necessary equipment, instrument, and logistical aspects of providing D&E. This allowed her to plan and organize a significant portion of the on-site clinical training. Although this may not always be possible, we recommend that interested trainees be offered the opportunity to at least observe if not participate in D&E clinical activities at an established site.

### Limitations

Our project has two important limitations. First, its success depended on the availability, dedication and motivation of two experienced gynecologists. Such providers may not exist or be available and willing learn D&E in other settings. Less experienced providers or mid-level providers may require longer training periods, making this partnership model less suitable. Another important limitation is that our project led to the introduction of D&E at only two hospitals in Brazil, which is not sufficient to meet the need of the Brazilian population. We will scale-up our effort to reach additional sites in the future, but the abortion landscape in Brazil is such that there are unfortunately very few sites that provide even medical abortion throughout the country. Women therefore often travel long distances to access these services. To improve abortion access throughout the country will require concerted efforts that go beyond scaling up our D&E training.

## Conclusions

We found that our three-phase capacity building project led to the successful introduction of D&E up to 16–18 weeks gestational age at two sites in Brazil. The project began as a partnership and with an invitation for a highly motivated OB-GYN to observe clinical activities at an established D&E site, followed by a focused clinical training period in Brazil. During that relatively short period we performed fewer proctored cases than we initially planned, but the two experienced gynecologists we trained were able to demonstrate competency with D&E in the early second trimester and have since performed procedures on their own without complications. We found that obtaining institutional buy-in and providing high quality didactics were important elements to ensure the success of our focused on-site training. We are hopeful that this D&E introduction project will eventually lead to the procedure being widely available as an option for Brazilian women and girls who need it.

## Data Availability Statement

The original contributions presented in the study are included in the article/supplementary material, further inquiries can be directed to the corresponding author.

## Ethics Statement

This project was approved by the Ethics Committee of Hospital Maternidade Vila Nova Cachoeirinha - Reference number 03/21, date: April 22, 2021. Written informed consent from the participants' legal guardian/next of kin was not required to participate in this study in accordance with the national legislation and the institutional requirements.

## Author Contributions

BS, SM, RR, HB, and NB contributed to the planning and implementation of the project. BS wrote the initial draft of the manuscript. RR and SM collected the patient characteristics and prepared the tables. All authors reviewed and approved the final version of the manuscript as submitted.

## Funding

The Society of Family Planning funded this project as BS' Fellowship in Family Planning Low Resource Setting Placement. There is no grant number available as the funder only provided reimbursements.

## Conflict of Interest

The authors declare that the research was conducted in the absence of any commercial or financial relationships that could be construed as a potential conflict of interest.

## Publisher's Note

All claims expressed in this article are solely those of the authors and do not necessarily represent those of their affiliated organizations, or those of the publisher, the editors and the reviewers. Any product that may be evaluated in this article, or claim that may be made by its manufacturer, is not guaranteed or endorsed by the publisher.
